# Supplementary material for: Discriminant Canonical Analysis of the Contribution of Spanish and Arabian Purebred Horses to the Genetic Diversity and Population Structure of Hispano-Arabian Horses
Source: Animals (Basel). 2021 Jan 21;11(2):269. doi: 10.3390/ani11020269 (PMC7912545; doi:10.3390/ani11020269)
Supplement: Supplementary file 1 [file animals-11-00269-s001.zip › Table S1.docx]

**Table S1**. Mean age (years) of the parents at the birth of their offspring for the four gametic routes in Spanish Purebred (PRE), Arabian Purebred (PRá) and Hispano-Arabian (Há) breeds.

| Parameter  Population set | Mean age of the parents at the birth of their offspring | Stallion  to colt | Mare to colt | Stallion to filly | Mare to filly | Total |
| --- | --- | --- | --- | --- | --- | --- |
| PRá | N | 10275 | 10210 | 11228 | 11180 | 42893 |
|  | Mean | 13.12 | 12.00 | 13.27 | 12.16 | 12.64 |
|  | SD | 13.35 | 12.23 | 13.77 | 12.98 | 13.12 |
| PRE | N | 83232 | 83154 | 89258 | 89160 | 344804 |
|  | Mean | 10.40 | 9.30 | 10.47 | 9.35 | 9.88 |
|  | SD | 6.17 | 5.40 | 6.45 | 5.82 | 6.00 |
| Há | N | 4262 | 4261 | 6353 | 6351 | 21227 |
|  | Mean | 11.55 | 11.68 | 20.97 | 21.16 | 17.27 |
|  | SD | 10.28 | 10.85 | 18.70 | 19.10 | 16.74 |
| SD: Standard deviation; SEM: Standard Error of the Mean. | | | | | | |
