# Supplementary material for: Discriminant Canonical Analysis of the Contribution of Spanish and Arabian Purebred Horses to the Genetic Diversity and Population Structure of Hispano-Arabian Horses
Source: Animals (Basel). 2021 Jan 21;11(2):269. doi: 10.3390/ani11020269 (PMC7912545; doi:10.3390/ani11020269)
Supplement: Supplementary file 1 [file animals-11-00269-s001.zip › Table S2.docx]

**Table S2.** Measures of genetic diversity, loss of genetic diversity, ancestors that explain 25%, 50% and 75% of the gene pool and average relatedness coefficient (ΔR).

| Breed | PRá | | PRE | | Há | |
| --- | --- | --- | --- | --- | --- | --- |
| Parameter/Population set | Historic | Current | Historic | Current | Historic | Current |
| Genetic diversity, GD (%) | 95.00 | 94.00 | 93.00 | 93.00 | 96.00 | 96.00 |
| Genetic diversity loss, GDL (%) | 5.00 | 6.00 | 7.00 | 7.00 | 4.00 | 4.00 |
| Genetic diversity in the reference population considered to compute the genetic diversity loss due to the unequal contribution of founders, DG* (%) | 99.00 | 99.00 | 98.00 | 98.00 | 98.00 | 99.00 |
| GDL due to genetic drift since founders (%) | 1.00 | 1.00 | 2.00 | 2.00 | 2.00 | 1.00 |
| GDL due to bottlenecks and genetic drift since founders (GL) (%) | 3.00 | 5.00 | 4.00 | 4.00 | 2.00 | 2.00 |
| GDL due to unequal founder contributions (%) | 5.00 | 6.00 | 7.00 | 7.00 | 4.00 | 4.00 |
| Ancestors explaining 25 % of the gene pool (n) | 3.00 | 2.00 | 2.00 | 2.00 | 3.00 | 3.00 |
| Ancestors explaining 50 % of the gene pool (n) | 9.00 | 6.00 | 5.00 | 5.00 | 8.00 | 8.00 |
| Ancestors explaining 75 % of the gene pool (n) | 54.00 | 27.00 | 14.00 | 14.00 | 24.00 | 24.00 |
| Average individual increase in inbreeding (ΔF) (%) | 1.00 | 1.00 | 1.00 | 1.00 | 1.00 | 1.00 |
| Average relatedness (AR) (%) | 1.0 | 11.00 | 4.00 | 1.00 | 11.00 | 4.00 |
