# Supplementary material for: Discriminant Canonical Analysis of the Contribution of Spanish and Arabian Purebred Horses to the Genetic Diversity and Population Structure of Hispano-Arabian Horses
Source: Animals (Basel). 2021 Jan 21;11(2):269. doi: 10.3390/ani11020269 (PMC7912545; doi:10.3390/ani11020269)
Supplement: Supplementary file 1 [file animals-11-00269-s001.zip › Table S3.docx]

**Table S3.** Summary of results for effective population size calculated from the individual inbreeding rate and through the individual coancestry rate and the number of equivalent subpopulations.

| Parameter  Breed | Population set | Effective population size calculated | | Number of equivalent subpopulations |
| --- | --- | --- | --- | --- |
|  |  | through individual inbreeding rate (N_e_F_i_) | through individual coancestry rate (N_e_C_i_) |  |
| PRá | Historic | 49.02 | 83.33 | 1.70 |
|  | Current | 43.86 | 69.44 | 1.58 |
| PRE | Historic | 49.02 | 8.98 | 0.18 |
|  | Current | 48.54 | 8.90 | 0.18 |
| Há | Historic | 52.63 | 24.27 | 0.46 |
|  | Current | 53.19 | 23.47 | 0.44 |
