# Supplementary material for: Discriminant Canonical Analysis of the Contribution of Spanish and Arabian Purebred Horses to the Genetic Diversity and Population Structure of Hispano-Arabian Horses
Source: Animals (Basel). 2021 Jan 21;11(2):269. doi: 10.3390/ani11020269 (PMC7912545; doi:10.3390/ani11020269)
Supplement: Supplementary file 1 [file animals-11-00269-s001.zip › Table S4.docx]

**Table S4.** Wright’s Fixation statistics; F_IS_ (inbreeding coefficient relative to the subpopulation), F_ST_ (Correlation between random gametes drawn from the subpopulation relative to the total population) and F_IT_ (Inbreeding coefficient relative to the total population).

| Parameter | Historic | Current |
| --- | --- | --- |
| F_IS_ (Inbreeding coefficient relative to the subpopulation) | 0.019 | 0.011 |
| F_ST_ (Correlation between random gametes drawn from the subpopulation relative to the total population) | 0.014 | 0.025 |
| F_IT_ (Inbreeding coefficient relative to the total population) | 0.033 | 0.036 |
| Mean inbreeding within subpopulations | 0.060 | 0.079 |
| Mean number of animals per subpopulation | 69033.333 | 54980.333 |
| Number of Nei genetic distances | 3 | 3 |
| Average Nei genetic distance | 0.013 | 0.025 |
| Mean coancestry within subpopulations | 0.062 | 0.049 |
| Selfcoancestry | 0.540 | 0.530 |
| Mean coancestry in the metapopulation | 0.048 | 0.025 |
| Subpopulations | 3 | 3 |
